# Pyruvate carboxylase and cancer progression

**DOI:** 10.1186/s40170-021-00256-7

**Published:** 2021-04-30

**Authors:** Violet A. Kiesel, Madeline P. Sheeley, Michael F. Coleman, Eylem Kulkoyluoglu Cotul, Shawn S. Donkin, Stephen D. Hursting, Michael K. Wendt, Dorothy Teegarden

**Affiliations:** 1 Department of Nutrition Sciences, Purdue University, West Lafayette, IN 47907 USA; 2 Department of Nutrition, University of North Carolina at Chapel Hill, Chapel Hill, USA; 3 Department of Medicinal Chemistry and Molecular Pharmacology, Purdue University, West Lafayette, USA; 4 Department of Animal Science, Purdue University, West Lafayette, USA; 5 Lineberger Comprehensive Cancer Center, University of North Carolina at Chapel Hill, Chapel Hill, USA

**Keywords:** Pyruvate carboxylase, Metastasis, Energy metabolism

## Abstract

Pyruvate carboxylase (PC) is a mitochondrial enzyme that catalyzes the ATP-dependent carboxylation of pyruvate to oxaloacetate (OAA), serving to replenish the tricarboxylic acid (TCA) cycle. In nonmalignant tissue, PC plays an essential role in controlling whole-body energetics through regulation of gluconeogenesis in the liver, synthesis of fatty acids in adipocytes, and insulin secretion in pancreatic β cells. In breast cancer, PC activity is linked to pulmonary metastasis, potentially by providing the ability to utilize glucose, fatty acids, and glutamine metabolism as needed under varying conditions as cells metastasize. PC enzymatic activity appears to be of particular importance in cancer cells that are unable to utilize glutamine for anaplerosis. Moreover, PC activity also plays a role in lipid metabolism and protection from oxidative stress in cancer cells. Thus, PC activity may be essential to link energy substrate utilization with cancer progression and to enable the metabolic flexibility necessary for cell resilience to changing and adverse conditions during the metastatic process.

## Background

Metabolic reprogramming is a notable hallmark of cancer and is critical for maintaining proliferation in cancer cells [1]. The enzyme pyruvate carboxylase (PC) contributes to cellular energy metabolism through converting pyruvate to oxaloacetate (OAA) and has been associated with metabolic reprogramming and increased tumor progression in a variety of cancer models [2–7]. In addition to its importance in replenishing the tricarboxylic acid (TCA) cycle to maintain energy production, the activity of PC is also important in fatty acid synthesis and generation of reducing equivalents for enhanced protection from oxidative stress [8, 9]. These functions provide unique abilities for cells to utilize a variety of energy substrates, depending on their availability, and places PC in a central role in enabling metabolic flexibility in cancer cells.

The primary metabolic pathways reprogrammed during cancer include glycolysis, glutaminolysis, and macromolecule synthesis, including nucleic acids, amino acids, and lipids. Increased activation of signaling pathways implicated in tumorigenesis such as PI3K/Akt/mTOR, c-Myc, and HIF-1 also results in a reprogrammed metabolic phenotype [10]. In order to survive and proliferate, cancer cells must balance increased needs for both macromolecule synthesis for daughter cell production and energy production under adverse conditions, such as nutrient-deficient environments. Thus, maximum flexibility to utilize a variety of energy substrates, including glucose, glutamine, and fatty acids, is essential for cancer progression with increasing evidence suggesting that cancer cells are reliant on reprogrammed metabolic pathways for progression through stages of cancer [10]. Thus, metabolic plasticity is critical throughout tumorigenesis for the survival of cancer cells in adverse microenvironments where varying levels of nutrients are available.

**Fig. 1:**
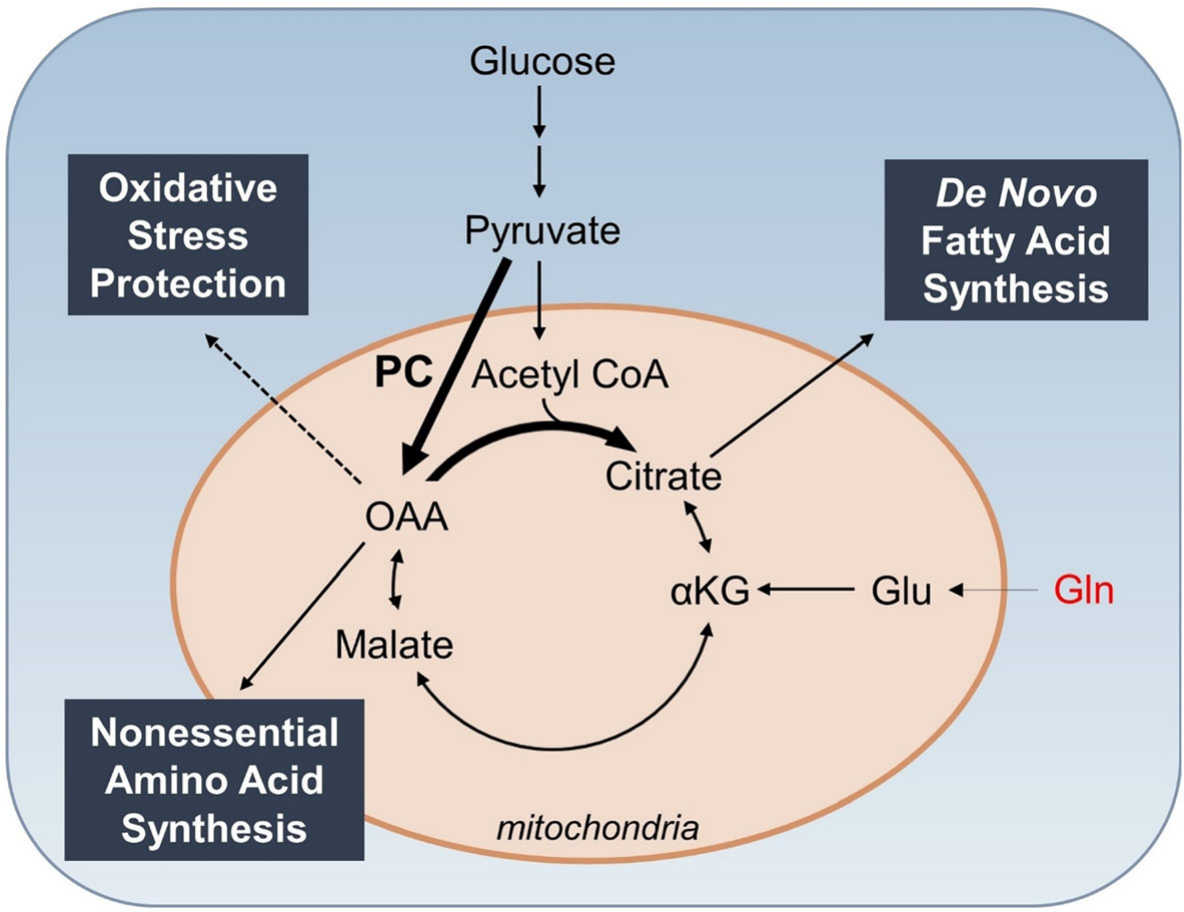
Pathways regulated by PC in cancer. PC plays a central role in metabolism and its regulation may contribute to metabolic plasticity in cancer cells, thereby increasing their metastatic potential. The PC reaction utilizes glucose-derived pyruvate to replenish OAA in the TCA cycle, which may be used for synthesis of fatty acids and/or amino acids when availability of these nutrients is limited. OAA produced from the PC reaction may also contribute to oxidative stress protection. Given the increased accumulation of ROS with cancer progression, the utilization of pyruvate in the PC reaction may be essential in conferring protection from cytotoxic levels of oxidative stress

Metabolic plasticity in cancer cells includes the ability to use available nutrients to support survival and growth in diverse and, in some cases, adverse conditions as cells metastasize [11]. Nutrient metabolism through the TCA cycle results in energy production and synthesis of metabolic intermediates, which are prerequisites to macromolecules required for cell proliferation [11, 12]. There are several main points of carbon entry into the TCA cycle in cells, including conversion of pyruvate to either OAA through the PC reaction or acetyl coenzyme A (acetyl-CoA) via pyruvate dehydrogenase. Another entry point is through glutamine anaplerosis, which results in the synthesis of the TCA cycle intermediate α-ketoglutarate (αKG) [13]. The ability to synthesize and utilize fatty acids is also important in the overall metabolic plasticity of cancer cells in progression, along with enhanced protection from oxidative stress, primarily resulting from increased metabolism [14]. Thus, PC has a potential role in mediating metabolic plasticity through the regulation of glucose metabolism, fatty acid synthesis, and generation of reducing equivalents to provide oxidative stress protection (Fig. 1). These functions of PC are a current topic of investigation in the field of cancer research.

## Roles and regulation of PC in nonmalignant tissues

The role and regulation of PC in metabolism have primarily been studied in the liver, adipose tissue, and pancreatic β cells, where PC contributes to maintaining glucose homeostasis, fatty acid synthesis, and insulin secretion, respectively [15]. Regulation of PC activity is consistent with its crucial role in regulating TCA cycle activity, gluconeogenesis, and overall energy homeostasis [12, 15–18]. Given its role in rapidly fluctuating glucose homeostasis, regulation of PC activity is orchestrated through both acute and long-term control.

Short-term positive allosteric regulation of PC activity by acetyl-CoA has been well characterized and allows for rapid responsiveness of TCA cycle capacity to substrate availability [19]. Acetyl-CoA promotion of PC activity results in increased generation of OAA to condense with acetyl-CoA to form citrate. Thus, in the presence of acetyl-CoA, adequate flux of OAA into the TCA cycle is maintained.

PC is also regulated through transcriptional regulation, and sustained changes in PC enzymatic activity require parallel increases in PC mRNA synthesis [20], PC mRNA tertiary structure, and PC translation rate [17]. The human PC gene is located on chromosome 11q13.2 and contains two alternative promoters, the distal (P2) and proximal (P1), that transcribe three separate transcript variants in humans [21]. The only difference in these transcript variants is their 5′-untranslated region, and as such, the coding sequence is identical for each variant [21]. In humans, the P1 promoter specifically regulates transcription of PC variant 2, and the P2 promoter initiates transcription of PC variants 1 and 3 [21]. A P3 promoter is unique to bovine [22]. Each promoter has distinct response elements, and the regulation by different promoter regions is dependent on organ type, allowing for differential expression. For example, PC in pancreatic β cells is under the control of the P2 promoter, while both the liver and adipose tissue express transcript variant 2 transcribed from the P1 promoter [21]. The regulation of expression by different promoter regions supports the variable roles of PC, dependent on the organ, such as fatty acid synthesis in adipocytes, regulation of glucose homeostasis in liver, and insulin secretion in the pancreatic β cells.

The function and regulation of PC expression during gluconeogenesis in the liver are well described [23], although unique aspects of control are still emerging across species. The hormone glucagon, which is elevated when glucose levels decline, induces the expression of PC in hepatocytes via a cAMP-responsive element (CRE) in the PC promoter [22]. Increased PC catalyzes the formation of OAA, the substrate of phosphoenolpyruvate carboxykinase (PCK), to drive hepatic gluconeogenesis and restore blood glucose. Genome-wide ChIP-based assay indicates that the PC promoter contains a CREB binding target site [24], and CREB upregulates PC expression [25]. Fatty acids also regulate PC expression [15, 17, 26, 27], although the direction and promoter activated depend on the fatty acid. For example, the activity of the P1 promoter in bovine is suppressed by stearic acid, whereas the P3 promoter is enhanced by stearic acid [26]. Furthermore, fatty acids that activate peroxisome proliferator-activated receptors (PPAR) appear to oppose the effects of stearic acid and promote PC expression [24]. Further evidence for regulation of PC by energy substrates in the liver is shown by the induction of PC mRNA by propionate in a concentration-dependent manner in primary hepatocytes [28]. There are also many examples where dietary energy intake regulates hepatic PC expression, including feed restriction in cows [29–31] and a decrease in expression in pregnant insulin-resistant rats fed a high-fat diet [32]. Thus, hepatic expression and activity of PC are regulated by alterations in cell and whole-body energy status and metabolism.

In adipocytes, PC is also essential to contribute to whole-body energy metabolism. PC provides OAA for the production of citrate in the mitochondria, which is transported to the cytosol and cleaved to form OAA and the fatty acid synthesis substrate, acetyl-CoA. In agreement with this function, PC expression increases dramatically during adipocyte differentiation [33, 34], is increased in adipocytes in obese animals, and is conversely reduced by physical activity in a murine model [35]. Consistent with its critical role in fatty acid synthesis in adipocytes, PPARγ, a master regulator of adipocyte fatty acid storage and fatty acid synthesis, positively regulates PC expression via a response element located in the P1 promoter [36, 37]. These results demonstrate PC’s function in fatty acid synthesis and regulation of energy metabolism in adipocytes.

PC expression and activity in pancreatic β cells primarily regulates insulin release in response to glucose. Transcriptional regulation of PC is mediated by the P2 promoter in pancreatic β cells, which contains response elements for both basal and pancreatic β cell-specific transcription factors. In pancreatic β cells, basal transcription activity is regulated by specificity protein 1/3 (Sp1/3) and nuclear transcription factor Y (NF-Y) [21]. Consistent with the ability of glucose to induce PC expression to promote insulin release, Pedersen et al. demonstrated a functional carbohydrate response element (ChoRE) in the distal promoter of the PC gene [21, 38]. In addition, pancreatic β cell-specific transcription factors such as pancreatic duodenal homeobox-1 (PDX1), musculoaponeurotic fibrosarcoma oncogene homolog A (v-Mafa1), and forkhead transcription factor boxA2 (Foxa2/HNF3β) also regulate PC expression, although direct activation is as yet not defined [17]. Further, the ubiquitously expressed proteins upstream stimulatory factors 1 and 2 (USF1 and USF2) upregulate PC expression through the P2 promoter [17, 21, 39]. In addition, a cholesterol sensor, the liver X receptor (LXR) [40] and the PPARα nuclear receptor have also been implicated in regulating PC expression in pancreatic β cells [41], although direct activation has not been shown as response elements have not been identified in the distal promoter region. These results demonstrate that a variety of factors regulate the expression of PC in pancreatic β cells to contribute to overall body glucose homeostasis.

PC also plays a pivotal role in nonmalignant brain tissue. Since pyruvate carboxylation is required for lipogenesis and neurotransmitter synthesis in astrocytes, defects in the expression or biotinylation of PC results in early death or a severe psychomotor retardation, which is manifested as a rare autosomal recessively inherited disease in early life [42]. Studies show that chemical changes in astrocytes can regulate PC expression. For example, increases in ammonia and ketone body concentration induce PC expression, whereas exogenous glutamate, anti-epileptic drugs, and DMSO decrease PC function via lowering biotinylation capacity of the enzyme [42].

Finally, PC also supports oxidative stress protection mechanisms through the generation of NADPH. OAA produced by PC can be converted to malate by malate dehydrogenase. Malate can be retained in the mitochondria to maintain the TCA cycle or may be transported to the cytosol. Decarboxylation of cytosolic malate to pyruvate via malic enzyme 1 (ME1) simultaneously reduces NADP^+^ to NADPH [43]. NADPH is critical for the conversion of oxidized glutathione (GSSG) to reduced glutathione (GSH), an antioxidant essential for the reduction of hydrogen peroxide (H_2_O_2_) to water [44]. Importantly, the pyruvate produced in the ME1 reaction can be converted back to OAA through PC and re-cycled through this pathway, a process termed pyruvate cycling [43]. Collectively, the contribution of PC to OAA pools to generate NADPH links PC to antioxidant defenses. As an experimental example, liver-specific PC knockout in mice impaired hepatic anaplerosis, depleted hepatic NADPH and glutathione, and increased oxidative stress [45]. These results support the link and importance of PC activity in antioxidant defenses in nonmalignant tissues.

## PC in cancer and metastasis

Metabolic reprogramming is a hallmark of cancer and is a potential target for cancer prevention and therapy [1]. The most notable example of reprogrammed metabolism in cancer cells is the Warburg effect, in which proliferating cancer cells favor the metabolism of glucose through aerobic glycolysis despite the presence of oxygen [46]. Cells with Warburg-like metabolism increase glucose consumption through glycolysis and upregulate flux of glucose-derived pyruvate through lactate dehydrogenase to route pyruvate to lactate production. Thus, Warburg-like metabolism provides cells with a ready supply of carbon backbones for compounds necessary for growth [47] and a robust energetic/reductive-oxidative (redox) buffer through pyruvate/lactate interconversion [48], while promoting a tumor permissive extracellular milieu [49]. While some cancers harbor genetic mutations which suppress mitochondrial ATP production, maintenance of substantial mitochondrial ATP production is generally selected for in cancer [47]. Hence, the result of Warburg-like metabolic reprogramming is increased glucose consumption and lactate production coupled with a proportional decreased flux of pyruvate into the TCA cycle, with both glycolysis and oxidative phosphorylation producing ATP. Increasing evidence suggests that anaplerosis of the pools of intermediate TCA cycle metabolites is essential for maintaining the cellular capacity for amino acid, nucleic acid, and lipid synthesis in proliferating cancer cells [50]. Given its critical role in TCA cycle anaplerosis, PC may therefore serve as a target to prevent cancer cell proliferation and cancer progression.

PC expression is upregulated in several types of cancer compared with normal tissue, including cancers of the mammary, lung, gallbladder, and papillary thyroid cancer [2–7]. For example, PC protein is overexpressed in human mammary cancer compared with normal mammary tissue and increases in abundance with tumor stage, as demonstrated by immunohistochemical analysis [5, 51]. In contrast, analysis of the METABRIC dataset failed to demonstrate significant differences in PC mRNA levels according to tumor stage [51]. However, analysis of this dataset revealed outlier groups bearing high-level PC expression within tumor stage groups. Increased PC expression in breast cancer is associated with reduced patient survival time [51], suggesting a role of PC in metastatic progression of cancer cells. While the mechanisms of PC upregulation in cancer remain to be definitively determined, PC lies within the 11q13.2 locus, a hotspot for gene amplification. Indeed, analysis of several patient datasets indicates that 16-30% of breast cancer patients harbor copy number gains in the PC gene in tumor tissue, and PC gene amplification corresponds with reduced survival [51]. Collectively, these results suggest a role of PC in cancer progression and increased mortality.

Cancer metastasis is a multistep process [52], and several of the steps involved likely require metabolic plasticity for successful transit and adaptation to the destination organ. Indeed, metastatic cancer cells display an increased requirement for ATP and redox defense, with prominent roles for glucose, fatty acid, and mitochondrial metabolism [53]. Thus PC, given its role in regulating glucose-derived anaplerotic carbon supply to the mitochondria, sits at a nexus of numerous metabolic pathways critical to metastasis. For instance, Shinde et al. showed that PC is required for mammary-to-lung metastasis in an in vivo model [51]. In this model, injection of 4T1 murine mammary cancer cells with genetic PC depletion into the mammary fat pads of BALB/c mice had no effect on primary tumor size, increased nonpulmonary metastasis, but dramatically decreased pulmonary metastases compared to PC-expressing cells [51]. These results indicate that PC is specifically required for tumor growth within the lungs. Consistent with these results, Christen et al. also observed an increase in PC expression and flux through PC in lung metastatic lesions of mice injected with 4T1 cells [54]. Furthermore, PC expression is required for growth of primary lung tumors [2]. These results are supported by mechanistic in vitro results showing that PC regulates processes specific to tumor cell growth within the lungs including overcoming oxidative stress [2, 5, 7, 51], and collectively suggest an organotropic role of PC in metastatic progression. In fact, Shinde et al. suggested that unique aspects of the pulmonary microenvironment, such as enhanced oxygenation and increased oxidative stress are the driving force requiring PC expression and activity in metastatic cells [51]. Indeed, both primary non-small cell lung cancer (NSCLC) tumors and breast cancer cells exhibit increased cysteine import and glutamate efflux to support redox balance [55, 56]. Further, nonessential amino acids become conditionally essential to support this process by supporting glutamate production via transamination [57]. Thus, loss of PC-mediated anaplerosis in the lung may promote increased demand for glutamate derived αKG to support TCA function.

The notion of PC-driven anaplerosis underpinning metastatic growth specifically in the lung is consistent with previous literature showing that metabolic reprogramming through modulation of specific enzymes is a requirement for cancer cell growth at specific sites. For example, the metabolic enzymes pyruvate dehydrogenase kinase 1 (PDK1) and PCK1 are required for hepatic colonization in animal models of breast and colon cancer, respectively [58, 59]. A better understanding of the mechanisms of PC expression and PC function in particular organ environments will help to refine contexts in which therapeutic targeting of PC might be most beneficial for preventing emergence of systemic disease.

## PC, energy metabolism, and cancer

Evidence from several models of cancer biology point to a critical role of PC in survival and progression of cancer. The mechanisms by which PC regulates cancer progression include modulation of glucose, lipid, and glutamine metabolism, as well as modulation of oxidative stress protection. Given PC’s central role as a regulator of each of these metabolic substrates, PC may confer metabolic plasticity to cancer cells, thereby improving survival of cancer cells in environments with variable nutrient availability.

### Glucose and the TCA cycle

Glucose is a primary source of carbon for energy production and biosynthesis in cancer cells. Increased flux of glucose-derived pyruvate into the TCA cycle through the PC reaction, providing energy and carbon backbones from glucose, appears to be a hallmark of certain cancers. The flux of glucose through metabolic pathways, including the TCA cycle, can be assessed using [^13^C]-labeled glucose and is useful in understanding alterations in cell metabolism with changing PC activity in cancers. For example, infusing NSCLC patients with universally labeled ^13^C glucose ([U-^13^C]-glucose) prior to tumor resection showed increased M+3 and M+5 labeling of aspartate, citrate, and malate, indicative of increased PC activity in tumor tissue compared to normal tissue [2, 6]. Likewise, an animal model of Kras^G12D^-driven NSCLC showed increased [U-^13^C]-glucose flux through PC in tumor tissue compared to non-tumor tissue [4]. Depletion of PC in this model led to impaired primary tumor formation, pointing to a requirement for PC in Kras^G12D^-driven NSCLC [4]. Furthermore, in A549 lung adenocarcinoma cells and MDA-MB-231 mammary cancer cells, PC depletion had no effect on glycolysis but decreased [U-^13^C]-glucose flux to aspartate, citrate, malate, and succinate [2, 60]. Finally, in 4T1 mammary cancer cells, PC depletion resulted in decreased glycolytic activity and oxygen consumption rate [51]. Taken together, these results demonstrate a critical anaplerotic role of PC in utilization of glucose metabolites in a spectrum of cancer models.

The role of PC in replenishing the TCA cycle intermediates in cancer models that are deficient in glucose or display impaired TCA cycle enzyme activity has been investigated extensively. For example, reduced expression of isocitrate dehydrogenase 1 (IDH1) in glioma models is associated with increased PC and decreased pyruvate dehydrogenase (PDH) activity [61]. In addition, PC plays a critical role in overcoming deficiencies in succinate dehydrogenase (SDH), an electron transport chain (ETC)/TCA complex that is commonly mutated in cancer [62–64]. The SDH complex is encoded by four genes (SDHA, SDHB, SDHC, SDHD) which couple with two assembly factors (SDHAF1 and SDHAF2) for function [65]. SDH converts succinate to fumarate in the TCA cycle, and inhibition of its activity can lead to depletion of downstream TCA intermediates. SDH ablation increases diversion of glucose to aspartate biosynthesis through PC activity in models of renal cell carcinoma [64]. Importantly, PC depletion reduces viability of renal cell carcinoma cells with SDH ablation, and addition of aspartate in vitro rescues cell growth in this model, suggesting that PC’s role in aspartate biosynthesis is crucial for overcoming SDH deficiency. Similarly, inhibition of SDHA or SDHB is sufficient to increase PC activity in prostate and neuroendocrine cancer cell models, leading to replenishment of cellular aspartate pools [62, 63]. Further, the use of the BRAF inhibitor, vemurafenib, in melanoma cells suppresses glycolysis but does not affect flux of glucose-derived pyruvate through PC, and results in an increase in the PC/PDH activity ratio. Addition of the PC inhibitor phenylacetic acid to vemurafenib-treated cells decreases cell growth, suggesting that blocking PC may sensitize melanoma cells to vemurafenib treatment [66, 67]. Given these data, PC’s role in replenishing the OAA pool for utilization in the TCA cycle or aspartate biosynthesis is crucial to consider when treating cells with therapies that target glucose metabolism. Cumulatively, these results support the importance of PC in replenishing the TCA cycle, which is critical in cancer cell progression.

### Lipid metabolism

Research from several types of cancer, including cancers of the mammary, colon, and prostate, show higher accumulation of lipids in cytoplasmic lipid droplets in more progressed cancers compared to early-stage cancers [68]. While the role of these cytoplasmic lipid droplets is poorly understood, their association with metastatic progression may highlight an important role of PC in promoting metastasis through fatty acid synthesis. De novo fatty acid synthesis is critical for biogenesis of cell membranes and signaling molecules and contributes to energy stored as triacylglycerol. The first step of de novo fatty acid synthesis is the carboxylation of cytoplasmic acetyl-CoA to form malonyl-CoA by acetyl-CoA carboxylase. ATP citrate lyase, the enzyme which cleaves cytoplasmic citrate into acetyl-CoA and OAA, therefore, enables fatty acid synthesis and connects carbohydrate metabolism to fatty acid biosynthesis. Thus, the initial role of PC in the synthesis of mitochondrial OAA, which is then used to synthesize citrate, is linked to fatty acid synthesis in the cytosol. Phannasil et al. demonstrate that MDA-MB-231 mammary cancer cells with genetic PC depletion showed decreased palmitate synthesis from [U-^13^C]-glucose compared to PC-expressing cells, highlighting the requirement of PC for fatty acid synthesis [60]. Similarly, suppression of PC in NSCLC cells decreased [U-^13^C]-glucose labeling only within the fatty acyl chains of phosphatidylcholine [2]. There was no ^13^C incorporation into the glycerol backbone of triacylglycerol, indicating that PC suppression reduces lipid synthesis specifically by inhibiting fatty acid synthesis. Interestingly, treating MCF10CA1a human mammary cancer cells with the hormone 1,25-dihydroxyvitamin D (1,25(OH)_2_D) resulted in decreased PC expression, suppressed synthesis of palmitate from glucose, and decreased triacylglycerol accumulation [8]. Overexpression of PC fully rescued palmitate synthesis and triacylglycerol accumulation in 1,25(OH)_2_D-treated cells, suggesting that 1,25(OH)_2_D mediates its regulation on fatty acid synthesis through suppression of PC [8]. Further, PC-mediated shunting of glucose-derived carbon to anabolic processes such as fatty acid synthesis limits NADH and ATP production from oxidative metabolism of glucose, both of which are negative regulators of glycolysis [14]. Thus, PC-mediated fatty acid synthesis may be of importance for maintaining high glycolytic activity in cancer cells, a process known to be crucial for cancer cell proliferation. Taken together, these results demonstrate the role of PC in supporting de novo fatty acid synthesis, a process which may be of therapeutic interest given the abundance of intracellular triacylglycerol in late-stage cancer cells.

### Glutamine and PC-driven anaplerosis

**Fig. 2:**
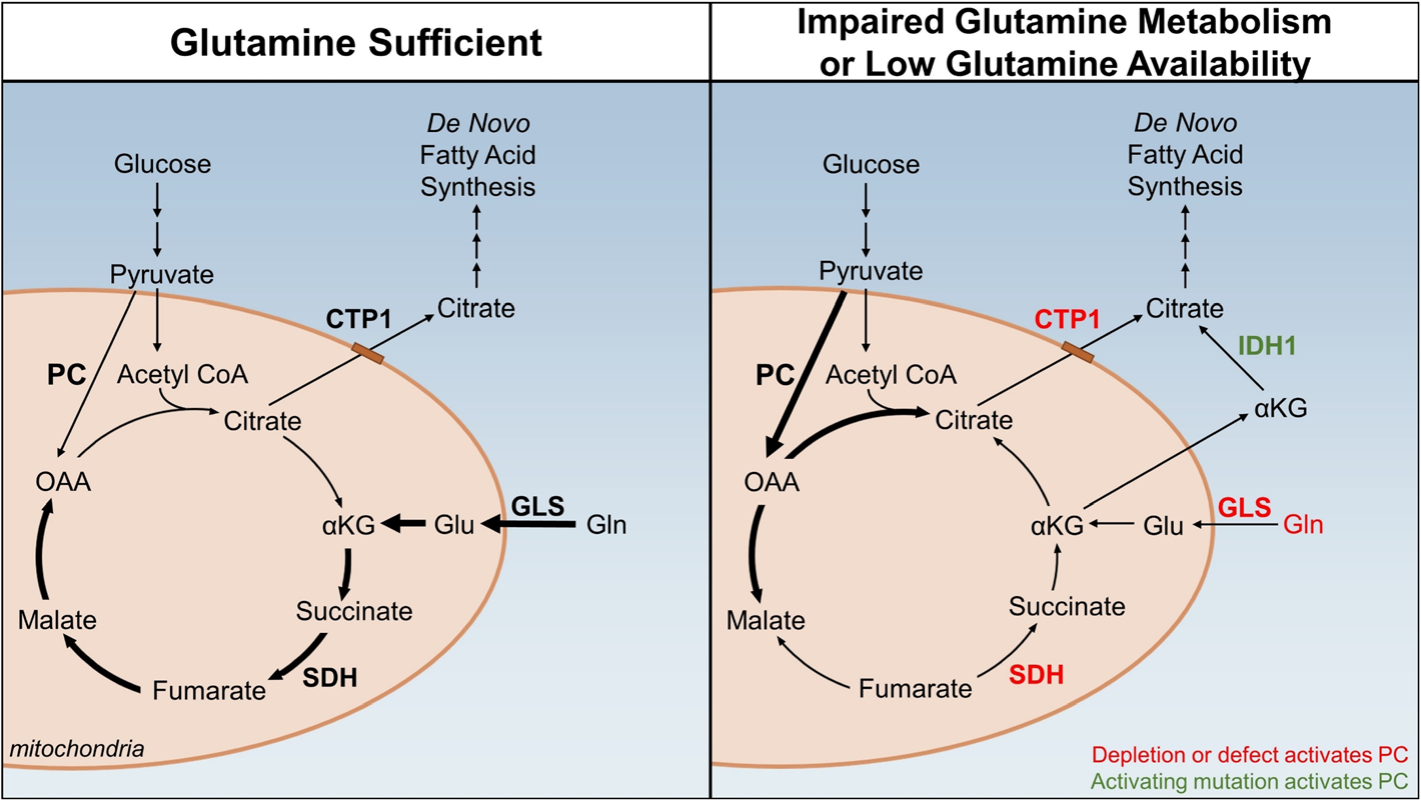
Cancer cells switch to PC-mediated anaplerosis when glutamine availability is limited. In glutamine-sufficient conditions (left panel), glutamine is converted to αKG to maintain pools of TCA cycle intermediates and their related biosynthetic reactions. When glutamine metabolism is impaired or glutamine availability is limited (right panel), cells may undergo a switch to PC-mediated anaplerosis which maintains TCA cycle intermediates under glutamine-depleted conditions. CTP, citrate transport protein; Glu, glutamate; Gln, glutamine; GLS, glutaminase; IDH1, isocitrate dehydrogenase 1; PDH, pyruvate dehydrogenase; SDH, succinate dehydrogenase

Glutamine is a critical anaplerotic substrate for the TCA cycle in proliferating cancer cells [13]. Evidence accumulated over the past decade, however, suggests that the anaplerotic demands of the cell can be fulfilled by OAA derived from the PC reaction in instances when glutamine availability is limited or is consumed for other processes (Fig. 2). Although glutamine is the most abundant amino acid in circulation, its availability may become growth-limiting for tumor cells due to the high metabolic demands of proliferating cancer cells for glutamine, the availability of glutamine in the local microenvironment of the tumor, or as a consequence of altered metabolism in cancer cells that prohibit flux of glutamine through αKG and the TCA cycle [13]. These factors collectively increase the cellular requirement for PC-driven TCA cycle anaplerosis.

Evidence supports the possibility of a proposed “switch” to PC-driven anaplerosis in cancer cells and related fibroblasts during glutamine depletion as a major component of cell adaptation to the tumor microenvironment. For example, glioblastoma cells grown in glutamine deprivation had increased flux of pyruvate through PC compared to cells grown in glutamine-replete conditions (Fig. 2) [69]. Human stromal prostate fibroblasts similarly failed to grow in glutamine-free media, however, activation of asparagine synthesis through a p62-Activating Transcription Factor 4 (ATF4)-PC signaling axis was sufficient to rescue cell growth, as asparagine can function as a nitrogen donor in glutamine depletion [70]. Importantly, asparagine production in fibroblasts also rescued growth of glutamine-starved cancer cells in a co-culture system [70], indicating that PC activation in stromal cells may play a critical role in maintaining viability of tumor cells. In addition to glutamine depletion, the ratio of available glutamine to pyruvate may also contribute to a switch to PC-mediated anaplerosis in cancer cells. The pyruvate/glutamine ratio was threefold higher in lung interstitial fluid compared to general circulation in healthy BALB/c mice, and metastatic mammary cancer cells in the lung microenvironment had increased PC activity compared to the primary tumor [54]. Adding pyruvate to cell culture media also increased PC activity in mammary cancer cells, collectively suggesting that increased availability of pyruvate relative to glutamine may be sufficient to switch cell metabolism to favor PC-mediated anaplerosis, particularly in the lung [54].

A switch to PC-driven anaplerosis is also shown in cells with impaired flux of glutamine into the TCA cycle. For example, blocking the flow of glutamine-derived carbon into the TCA cycle through depletion of glutaminase increased PC activity in glioblastoma cells as measured by [1-^13^C]-pyruvate flux [69]. Flux of pyruvate through PC was similarly increased in glioma cells containing an isocitrate dehydrogenase 1 (IDH1) mutation in which glutamine-derived αKG is preferentially converted to 2-hydroxyglutarate, leading to depletion of TCA cycle intermediates [61]. Further evidence for PC compensating for glutamine is shown as depletion of citrate transport protein (CTP), which limits entry of glutamine-derived αKG into the TCA cycle, in H460 large cell lung cancer cells also increased PC activity [71]. The importance of PC to replenish the TCA cycle is also demonstrated in cells carrying SDH mutations that block flow of glutamine-derived αKG through the TCA cycle, leading to an increase in PC activity that replenishes metabolites downstream of the SDH reaction [62]. In sum, these data support that cancer cells undergo a switch to PC-driven anaplerosis when flux of glutamine through the TCA cycle is impaired.

### Oxidative stress

Reactive oxygen species (ROS) are abundant in cancer cells and are produced as byproducts of metabolism [72], and PC activity is implicated in oxidative stress protection in cancer cells.

For example, 4T1 mammary cancer cells with constitutive PC depletion are significantly more sensitive to H_2_O_2_ compared to their PC-expressing counterparts [51]. Further, inhibition of PC via a doxycycline-inducible shRNA construct decreased NADPH/NADP+ and GSH/GSSG ratios, increased intracellular ROS, and increased sensitivity to H_2_O_2_ treatment in MCF10A-*ras* mammary cancer cells compared to untreated control cells [9]. Similarly, GSH synthesis is reduced when PC is depleted in NSCLC cells [2]. These results suggest that loss of PC impedes production of reducing agents required for protection from intracellular ROS. In sum, PC plays a role in protection from ROS in mammary cancer cells and may contribute to oxidative stress protection.

Consistent with a potential role of PC in protection from ROS, increased ROS may increase PC activity. For example, treating A549 NSCLC cells with selenite, an inorganic form of selenium, increased ROS and PC activity [73]. Further, treating K562 acute myeloid leukemia cells with bezafibrate and medroxyprogesterone (BaP), a known inducer of ROS, similarly increased PC activity [74]. These data may collectively suggest that ROS stimulates PC activity; however, the mechanism of this regulation has not been defined.

## Regulation of PC in cancer cells

**Fig. 3:**
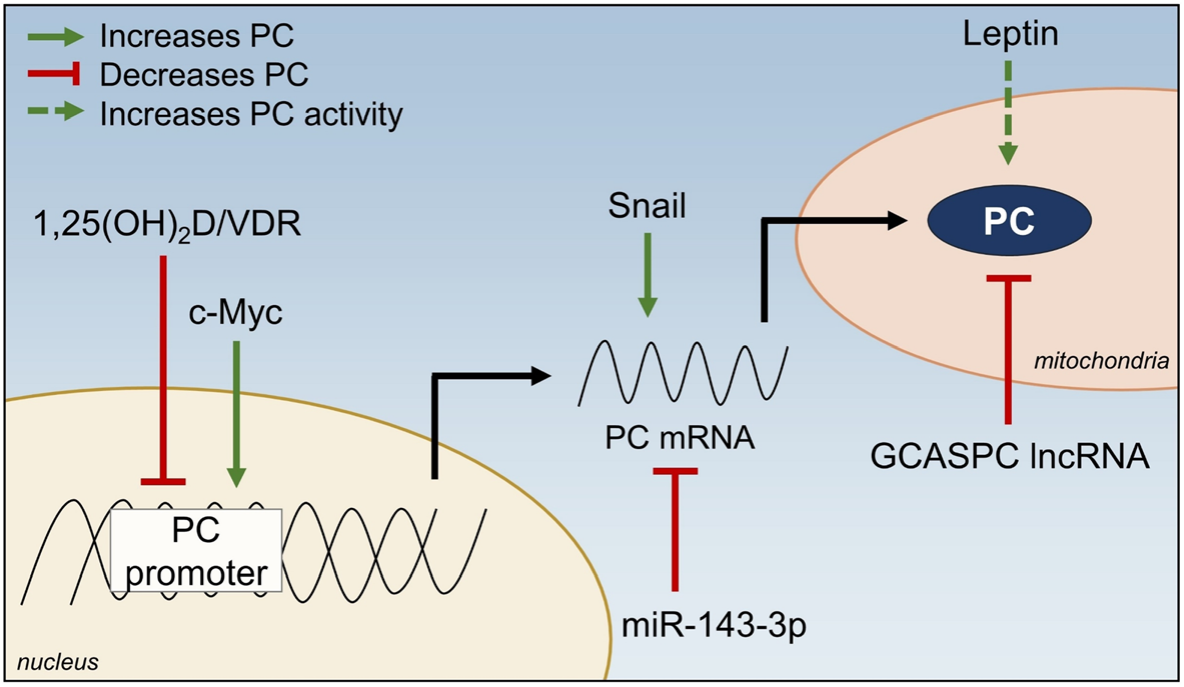
Regulation of PC in cancer cells. Several factors have been identified which modify PC transcriptional activation, transcript levels, and protein levels specifically in cancer cells. Further elucidation of PC regulators in the context of cancer may be useful in identifying strategies to inhibit PC and block cancer progression. 1,25(OH)_2_D—1,25-dihydroxyvitamin D; GCASPC lncRNA—gallbladder cancer-associated suppressor of pyruvate carboxylase long noncoding RNA; VDR—vitamin D receptor

Given the requirement of PC for progression, understanding the mechanisms that regulate expression and activity of PC in cancer cells is important to identify targets to prevent metastasis. Several mechanisms of PC regulation have been identified in cancer models, including regulation by non-coding RNAs, signaling pathways, and small molecules, further demonstrating the centrality of PC in metastasis (Fig. 3).

PC is regulated by non-coding RNAs, including long non-coding- and micro-RNAs (Fig. 3). Cell and patient-derived tumor tissue models of gallbladder cancer demonstrated the importance of the long non-coding RNA (lncRNA) gallbladder cancer-associated suppressor of pyruvate carboxylase (GCASPC) in regulation of PC expression [3]. Immunoprecipitation studies support that GCASPC lncRNA physically interacts with PC protein in the mitochondria leading to its degradation, suggesting that the loss of GCASPC lncRNA that occurs in gallbladder cancer may lead to increased PC protein retention. Overexpression of GCASPC lncRNA decreased PC protein expression and activity, cell proliferation, and tumor growth in vivo, and proliferation was rescued when PC was overexpressed. These data highlight the role of PC in primary tumor formation and regulation by GCASPC lncRNA. In MDA-MB-231 mammary cancer cells, overexpression of the microRNA miR-143-3p resulted in decreased PC expression, cell proliferation, and migration [75]. A luciferase reporter assay confirmed the direct binding of miR-143-3p to the 3′ untranslated region of PC mRNA, resulting in decreased PC mRNA and protein expression. However, re-expression of PC in miR-143-3p-overexpressing MDA-MB-231 cells increased PC protein expression but did not overcome the inhibitory effects of miR-143-3p on proliferation and migration, suggesting inhibition of PC is only partially responsible for the antiproliferative effects of miR-143-3p on mammary cancer cells. Taken together, these studies demonstrate PC regulation in cancer by non-coding RNAs, which may be novel targets for inhibiting PC in conjunction with other cancer treatments.

Cancer-promoting signaling pathways also play a role in modifying PC expression (Fig. 3). In OVCAR-3 ovarian cancer cells, activity of tankyrase enzyme, a member of the poly(ADP-ribose) polymerase (PARP) family, maintains PC expression through Wnt/β-catenin/Snail signaling [76]. Similar increases in PC protein expression through Wnt/β-catenin/Snail signaling were observed in MCF-7 mammary cancer cells [77]. Both studies demonstrated that Snail was required for PC expression, although the mechanism by which Snail stabilizes PC has not been elucidated [76, 77]. Stage and cell type may play a role in Snail’s ability to regulate PC, as overexpression of Snail in MCF10A-*ras* cells, which represent an early model of mammary cancer, did not result in an increase in PC expression [51]. c-Myc may be an additional signaling molecule that promotes PC expression, as a positive association between c-Myc and PC was found in transcriptome data from The Cancer Genome Atlas (TCGA) breast cancer dataset [78]. Further investigation demonstrated that inhibition of c-Myc decreased PC expression in MDA-MB-231 cells through a direct mechanism, but had no effect on PC expression in MCF-7 human mammary cancer cells [78]. Further, inhibition of c-Myc in MDA-MB-231 cells decreased cell viability, migration, and invasion. PC overexpression partly rescued viability of c-Myc-inhibited cells, but PC overexpression did not rescue cell migration or invasion, suggesting that c-Myc-mediated regulation of PC plays an important role in cell viability but not migration or invasion, at least in the MDA-MB-231 breast cancer cell model [78]. Given that PC expression is associated with cancer progression, it is not surprising that these data indicate regulation of PC expression by signaling pathways that are commonly upregulated in cancer.

Finally, hormones, including leptin and the hormonal form of vitamin D, are regulators of PC (Fig. 3). The bioactive metabolite of vitamin D, 1,25(OH)_2_D, decreases PC expression in non-metastatic human MCF10A-*ras* mammary cancer cells [9] and in metastatic human MCF10CA1a cells [8]. In MCF10A-*ras* cells, 1,25(OH)_2_D directly decreases PC expression through a vitamin D response element (VDRE) in the promoter region of the PC gene [9]. Further evidence of a VDRE in the distal promoter of the human PC gene was identified in MDA-MB-231 metastatic mammary cancer cells [76]. Interestingly, treatment of MCF-7 cells with leptin, a hormone secreted by adipocytes, increases PC activity as well as the ratio of PC/PDH activity [79]. Increased circulating leptin is associated with obesity and increased cancer risk [80]; thus, leptin-mediated regulation of PC activity is particularly interesting, considering the increased mortality from cancer observed in obese individuals [81]. Given the prevalence of vitamin D deficiency [82] and the association of low vitamin D status with obesity [83], these studies illuminate a potential strategy to reduce PC activity with vitamin D in PC-dependent cancers.

## Small molecule inhibitors of PC

Given the importance of PC in cancer progression and other metabolic disease such as diabetes, several studies have explored means to therapeutically inhibit PC activity. PC catalyzes the carboxylation of pyruvate in two steps. First, biotin covalently attaches to an N-terminal lysine residue in the PC protein. This biotin becomes carboxylated in an ATP-dependent fashion. The carboxybiotin acts as a mobile carrier by moving to the carboxyl transferase domain of PC where the carboxyl group is transferred onto pyruvate to form oxaloacetate [84]. Given the need for biotin in this reaction, avidin acts as a potent inhibitor of PC, and in vitro experiments using avidin with PC have led to numerous advances in our understanding of the regulatory mechanisms, stoichiometry, and structure-function relationships of the enzyme. It is important to note that avidin-based PC inhibition has many off-target effects, specifically in pancreatic β-cells of insulin-resistant murine models [85, 86]. Therefore, avidin-based molecules are not likely to translate into clinical therapeutics. In addition to avidin, several other molecules have been found to non-specifically regulate the activity of PC, including various nucleosides and pyruvate derivatives. Additionally, there are small molecules that bind PC outside of the carboxyl transferase domain within an allosteric site. In particular, aspartate, glutamate, and αKG can act as allosteric inhibitors of PC, providing regulatory feedback to control the TCA cycle [87]. Overall, different allosteric inhibitors deactivate a metabolic compensation mechanism called glucose-stimulated insulin secretion (GSIS) in pancreatic cells of pre-diabetic/diabetic cancer patients [88].

Despite this detailed knowledge of the mechanisms and regulation of PC activity, there is a paucity of small molecules capable of target-specific inhibition of the enzyme. A recent study developed ZY-444, a small molecule which appears to bind to PC and inhibit enzymatic activity [89]. The exact binding mode of ZY-444 to PC was not determined, and several off-target binding proteins were identified. However, use of this molecule in vitro and in vivo phenocopied results obtained with genetic depletion of PC, and PC expression was necessary for ZY-444 to elicit an impact on metabolism. Continued advances in the pharmacological targeting of PC will be required to properly assess modulation of PC activity as a therapeutic approach for cancer and other diseases. Additionally, Kumashiro et al. developed antisense oligonucleotides (ASO) to achieve in vivo depletion of PC expression. This study found that a PC-targeted ASO-reduced PC expression in the liver and adipose tissue, leading to decreased plasma glucose and lipid concentrations, decreased adiposity, and suppression of endogenous glucose production in pre-diabetic rats [90]. Overall, while these genetic and chemical targeting approaches suggest that inhibition of PC in cancer cells can block cancer progression, current approaches lack tissue specificity. Therefore, strategies to localize PC inhibitors to metastatic tumors are needed to avoid systemic toxicities and maximize anti-tumor efficiency [90].

## Conclusions

Overall, previous literature provides support of a crucial role of PC-mediated anaplerosis in the growth and survival of cancer cells that may be critical for the metabolic flexibility necessary for metastasis. Several studies have shown a requirement for PC expression for specific steps in metastasis in vitro and in vivo [2, 4, 5, 7, 51, 64] and an essential role in mammary to pulmonary metastasis [51]. PC has a central role in regulating energy metabolism, coordinating glucose, glutamine, and lipid metabolism. This central role of PC in regulating energy metabolism may increase cellular metabolic plasticity that is required to adapt to changes in nutrient availability during cancer growth and progression (Fig. 1). The role of PC in coordinating energy metabolism may be particularly relevant in the pulmonary microenvironment, as PC expression and activity may drive the growth of metastatic cells at this site [51]. In addition, PC may play a role in conferring resistance to therapies [66, 67], suggesting that inhibition of PC in human cancers may be a useful form of adjuvant treatment to improve patient outcomes. To fully understand the potential role of PC to function as a regulator of metabolic plasticity, it is important to investigate its effects in specific stages of cancer progression and under physiologically relevant conditions. Future studies linking PC and cancer progression using in vivo model systems should include measures of glucose, glutamine, and pyruvate concentrations, as well as the stage of cancer progression and the tumor microenvironment in vivo.

Several potential mechanisms by which PC-mediated anaplerosis may increase cancer cell survival have been elucidated (Fig. 1). PC plays a critical role in replenishing TCA cycle intermediates that are required for cell proliferation, particularly in the context of glutamine deprivation or impaired glutamine utilization [54, 61, 62, 69, 71]. In addition, PC-mediated fatty acid synthesis and oxidative stress protection have been demonstrated in a variety of models [2, 9, 51, 60, 74]. These two effects of PC activity are of particular interest in the context of cancer progression. Accumulation of lipids from upregulated fatty acid synthesis are features of progressed stages of cancer in vitro and in vivo [8, 91]. Increased PC activity promotes the synthesis of citrate, which is used in de novo fatty acid synthesis, and further promotes cancer cell growth and progression. Cancer cells are also exposed to increasing levels of oxidative stress throughout progression [92], suggesting that protection conferred by PC expression is critical for metastatic progression. The interaction between ROS production, PC expression and activity, fatty acid metabolism, and oxidative stress protection is an interesting avenue for further investigation.

Current evidence suggests that PC may be required for specific steps of the metastatic cascade. For example, genetic depletion of PC suppresses migration and invasion in breast cancer cells [5], suggesting that PC may play an important role in enabling metastatic outgrowth from the primary tumor into the surrounding tissue. Further, PC is critical in oxidative stress protection [9], and thus, its activity may be essential for survival in metastatic processes with high levels of oxidative stress [93, 94]. Utilizing inducible shPC constructs in vitro and in xenograft cancer models may offer an avenue to investigate the specific steps at which PC is required for metastatic progression, which will aid in determining stages at which PC-targeted therapies may be most effective in a clinical setting.

In sum, the accumulating evidence on the role of PC in cancer over the past decade suggests that PC, via its regulatory effects on multiple energy pathways, plays a central role in providing the metabolic flexibility necessary for cancer progression and metastasis. Future research should focus on PC as a potential intervention target for preventing cancer progression.

## Data Availability

Not applicable.

## Abbreviations

1,25(OH)_2_D: 1,25-Dihydroxyvitamin D
αKG: α-Ketoglutarate
Acetyl-CoA: Acetyl coenzyme A
ASO: Antisense oligonucleotides
ATF4: Activating transcription factor 4
BaP: Bezafibrate and medroxyprogesterone
ChoRE: Carbohydrate response element
CRE: cAMP-responsive element
CTP: Citrate transport protein
ETC: Electron transport chain
Foxa2/HNF3β: Forkhead transcription factor boxA2
GCASPC: Gallbladder cancer-associated suppressor of pyruvate carboxylase
GSH: Reduced glutathione
GSSG: Oxidized glutathione
H_2_O_2_: Hydrogen peroxide
IDH: Isocitrate dehydrogenase
lncRNA: Long non-coding RNA
LXR: Liver X receptor
ME1: Malic enzyme 1
NF-Y: Nuclear transcription factor Y
NSCLC: Non-small cell lung cancer
OAA: Oxaloacetate
PARP: Poly(ADP-ribose)
PC: Pyruvate carboxylase
PCK: Phosphoenolpyruvate carboxykinase
PDH: Pyruvate dehydrogenase
PDK1: Pyruvate dehydrogenase kinase 1
PDX1: Pancreatic duodenal homeobox-1
PPAR: Peroxisome proliferator-activated receptors
redox: Oxidation-reduction
ROS: Reactive oxygen species
SDH: Succinate dehydrogenase
Sp1/3: Specificity protein 1/3
TCA: Tricarboxylic acid
TCGA: The Cancer Genome Atlas
USF: Upstream stimulatory factors
[U-^13^C]-glucose: Universally labeled ^13^C glucose
v-Mafa1: Musculoponeurotic fibrosarcoma oncogene homolog A
VDRE: Vitamin D response element
